# Supplementary material for: Pyruvate Dehydrogenase Kinase 1 inhibition mediated oxidative phosphorylation enhancement in cartilage promotes osteoarthritis progression
Source: BMC Musculoskelet Disord. 2023 Jul 20;24:597. doi: 10.1186/s12891-023-06585-6 (PMC10357736; doi:10.1186/s12891-023-06585-6)
Supplement: Supplementary file 1 — Additional file 1. [file 12891_2023_6585_MOESM1_ESM.zip › KEGG.Gsea.1653622432464/KEGG_VIRAL_MYOCARDITIS.html]

Details for gene set KEGG\_VIRAL\_MYOCARDITIS[GSEA]

|  || Dataset | 55457\_collapsed\_to\_symbols.cls.cls #OA\_versus\_normal.cls.cls #OA\_versus\_normal\_repos |
| Phenotype | cls.cls#OA\_versus\_normal\_repos |
| Upregulated in class | normal |
| GeneSet | KEGG\_VIRAL\_MYOCARDITIS |
| Enrichment Score (ES) | -0.20893851 |
| Normalized Enrichment Score (NES) | -0.83899957 |
| Nominal p-value | 0.7607573 |
| FDR q-value | 0.9034906 |
| FWER p-Value | 1.0 |
Table: GSEA Results Summary

  

Fig 1: Enrichment plot: KEGG\_VIRAL\_MYOCARDITIS      
 Profile of the Running ES Score & Positions of GeneSet Members on the Rank Ordered List

  

| SYMBOL | TITLE | RANK IN GENE LIST | RANK METRIC SCORE | RUNNING ES | CORE ENRICHMENT || 1 | SGCA | sarcoglycan alpha [Source:HGNC Symbol;Acc:HGNC:10805] | 196 | 0.824 | 0.0260 | Yes |
| 2 | HLA-DMB | "major histocompatibility complex, class II, DM beta [Source:HGNC Symbol;Acc:HGNC:4935]" | 679 | 0.598 | 0.0185 | Yes |
| 3 | SGCB | sarcoglycan beta [Source:HGNC Symbol;Acc:HGNC:10806] | 687 | 0.597 | 0.0479 | Yes |
| 4 | CD86 | CD86 molecule [Source:HGNC Symbol;Acc:HGNC:1705] | 689 | 0.596 | 0.0777 | Yes |
| 5 | ITGB2 | integrin subunit beta 2 [Source:HGNC Symbol;Acc:HGNC:6155] | 827 | 0.562 | 0.0952 | Yes |
| 6 | SGCG | sarcoglycan gamma [Source:HGNC Symbol;Acc:HGNC:10809] | 1284 | 0.461 | 0.0827 | Yes |
| 7 | HLA-DQA1 | "major histocompatibility complex, class II, DQ alpha 1 [Source:HGNC Symbol;Acc:HGNC:4942]" | 1334 | 0.452 | 0.1016 | Yes |
| 8 | MYH13 | myosin heavy chain 13 [Source:HGNC Symbol;Acc:HGNC:7571] | 1561 | 0.419 | 0.1050 | Yes |
| 9 | CD55 | CD55 molecule (Cromer blood group) [Source:HGNC Symbol;Acc:HGNC:2665] | 1619 | 0.411 | 0.1211 | Yes |
| 10 | CXADR | CXADR Ig-like cell adhesion molecule [Source:HGNC Symbol;Acc:HGNC:2559] | 1719 | 0.395 | 0.1332 | Yes |
| 11 | HLA-DMA | "major histocompatibility complex, class II, DM alpha [Source:HGNC Symbol;Acc:HGNC:4934]" | 1769 | 0.388 | 0.1489 | Yes |
| 12 | SGCD | sarcoglycan delta [Source:HGNC Symbol;Acc:HGNC:10807] | 1781 | 0.387 | 0.1674 | Yes |
| 13 | HLA-DOB | "major histocompatibility complex, class II, DO beta [Source:HGNC Symbol;Acc:HGNC:4937]" | 2147 | 0.344 | 0.1563 | Yes |
| 14 | MYH1 | myosin heavy chain 1 [Source:HGNC Symbol;Acc:HGNC:7567] | 2297 | 0.327 | 0.1610 | Yes |
| 15 | MYH2 | myosin heavy chain 2 [Source:HGNC Symbol;Acc:HGNC:7572] | 2459 | 0.307 | 0.1639 | Yes |
| 16 | MYH6 | myosin heavy chain 6 [Source:HGNC Symbol;Acc:HGNC:7576] | 2555 | 0.297 | 0.1714 | Yes |
| 17 | HLA-DQB1 | "major histocompatibility complex, class II, DQ beta 1 [Source:HGNC Symbol;Acc:HGNC:4944]" | 2840 | 0.266 | 0.1626 | Yes |
| 18 | MYH8 | myosin heavy chain 8 [Source:HGNC Symbol;Acc:HGNC:7578] | 3119 | 0.238 | 0.1529 | Yes |
| 19 | MYH7 | myosin heavy chain 7 [Source:HGNC Symbol;Acc:HGNC:7577] | 3161 | 0.234 | 0.1614 | Yes |
| 20 | CASP3 | caspase 3 [Source:HGNC Symbol;Acc:HGNC:1504] | 3202 | 0.230 | 0.1698 | Yes |
| 21 | DAG1 | dystroglycan 1 [Source:HGNC Symbol;Acc:HGNC:2666] | 3203 | 0.230 | 0.1814 | Yes |
| 22 | RAC3 | Rac family small GTPase 3 [Source:HGNC Symbol;Acc:HGNC:9803] | 3849 | 0.171 | 0.1397 | No |
| 23 | MYH10 | myosin heavy chain 10 [Source:HGNC Symbol;Acc:HGNC:7568] | 4169 | 0.141 | 0.1219 | No |
| 24 | HLA-DOA | "major histocompatibility complex, class II, DO alpha [Source:HGNC Symbol;Acc:HGNC:4936]" | 4344 | 0.125 | 0.1146 | No |
| 25 | CD28 | CD28 molecule [Source:HGNC Symbol;Acc:HGNC:1653] | 4543 | 0.110 | 0.1046 | No |
| 26 | EIF4G3 | eukaryotic translation initiation factor 4 gamma 3 [Source:HGNC Symbol;Acc:HGNC:3298] | 4675 | 0.098 | 0.0993 | No |
| 27 | MYH4 | myosin heavy chain 4 [Source:HGNC Symbol;Acc:HGNC:7574] | 4702 | 0.096 | 0.1021 | No |
| 28 | MYH7B | myosin heavy chain 7B [Source:HGNC Symbol;Acc:HGNC:15906] | 5024 | 0.071 | 0.0807 | No |
| 29 | MYH11 | myosin heavy chain 11 [Source:HGNC Symbol;Acc:HGNC:7569] | 5211 | 0.056 | 0.0690 | No |
| 30 | HLA-DPB1 | "major histocompatibility complex, class II, DP beta 1 [Source:HGNC Symbol;Acc:HGNC:4940]" | 5486 | 0.033 | 0.0493 | No |
| 31 | CD40LG | CD40 ligand [Source:HGNC Symbol;Acc:HGNC:11935] | 5529 | 0.030 | 0.0475 | No |
| 32 | DMD | dystrophin [Source:HGNC Symbol;Acc:HGNC:2928] | 5605 | 0.023 | 0.0429 | No |
| 33 | BID | BH3 interacting domain death agonist [Source:HGNC Symbol;Acc:HGNC:1050] | 5715 | 0.014 | 0.0351 | No |
| 34 | MYH15 | myosin heavy chain 15 [Source:HGNC Symbol;Acc:HGNC:31073] | 5874 | 0.004 | 0.0230 | No |
| 35 | RAC2 | Rac family small GTPase 2 [Source:HGNC Symbol;Acc:HGNC:9802] | 6098 | -0.013 | 0.0062 | No |
| 36 | HLA-DRA | "major histocompatibility complex, class II, DR alpha [Source:HGNC Symbol;Acc:HGNC:4947]" | 6550 | -0.046 | -0.0266 | No |
| 37 | CD40 | CD40 molecule [Source:HGNC Symbol;Acc:HGNC:11919] | 6825 | -0.065 | -0.0447 | No |
| 38 | CD80 | CD80 molecule [Source:HGNC Symbol;Acc:HGNC:1700] | 6918 | -0.074 | -0.0482 | No |
| 39 | HLA-DRB1 | "major histocompatibility complex, class II, DR beta 1 [Source:HGNC Symbol;Acc:HGNC:4948]" | 7087 | -0.086 | -0.0570 | No |
| 40 | RAC1 | Rac family small GTPase 1 [Source:HGNC Symbol;Acc:HGNC:9801] | 7192 | -0.096 | -0.0603 | No |
| 41 | HLA-F | "major histocompatibility complex, class I, F [Source:HGNC Symbol;Acc:HGNC:4963]" | 7196 | -0.096 | -0.0557 | No |
| 42 | CAV1 | caveolin 1 [Source:HGNC Symbol;Acc:HGNC:1527] | 7205 | -0.097 | -0.0515 | No |
| 43 | LAMA2 | laminin subunit alpha 2 [Source:HGNC Symbol;Acc:HGNC:6482] | 7392 | -0.110 | -0.0605 | No |
| 44 | CCND1 | cyclin D1 [Source:HGNC Symbol;Acc:HGNC:1582] | 7512 | -0.119 | -0.0638 | No |
| 45 | MYH14 | myosin heavy chain 14 [Source:HGNC Symbol;Acc:HGNC:23212] | 8032 | -0.158 | -0.0963 | No |
| 46 | CASP9 | caspase 9 [Source:HGNC Symbol;Acc:HGNC:1511] | 8142 | -0.166 | -0.0965 | No |
| 47 | MYH3 | myosin heavy chain 3 [Source:HGNC Symbol;Acc:HGNC:7573] | 8258 | -0.176 | -0.0966 | No |
| 48 | PRF1 | perforin 1 [Source:HGNC Symbol;Acc:HGNC:9360] | 8265 | -0.176 | -0.0882 | No |
| 49 | CYCS | "cytochrome c, somatic [Source:HGNC Symbol;Acc:HGNC:19986]" | 8410 | -0.186 | -0.0901 | No |
| 50 | MYH9 | myosin heavy chain 9 [Source:HGNC Symbol;Acc:HGNC:7579] | 8463 | -0.191 | -0.0846 | No |
| 51 | ABL1 | "ABL proto-oncogene 1, non-receptor tyrosine kinase [Source:HGNC Symbol;Acc:HGNC:76]" | 9081 | -0.238 | -0.1207 | No |
| 52 | HLA-DPA1 | "major histocompatibility complex, class II, DP alpha 1 [Source:HGNC Symbol;Acc:HGNC:4938]" | 9481 | -0.273 | -0.1382 | No |
| 53 | ITGAL | integrin subunit alpha L [Source:HGNC Symbol;Acc:HGNC:6148] | 9526 | -0.276 | -0.1278 | No |
| 54 | ICAM1 | intercellular adhesion molecule 1 [Source:HGNC Symbol;Acc:HGNC:5344] | 9740 | -0.296 | -0.1295 | No |
| 55 | FYN | "FYN proto-oncogene, Src family tyrosine kinase [Source:HGNC Symbol;Acc:HGNC:4037]" | 10092 | -0.330 | -0.1403 | No |
| 56 | CASP8 | caspase 8 [Source:HGNC Symbol;Acc:HGNC:1509] | 10442 | -0.366 | -0.1491 | No |
| 57 | HLA-G | "major histocompatibility complex, class I, G [Source:HGNC Symbol;Acc:HGNC:4964]" | 10869 | -0.413 | -0.1616 | No |
| 58 | EIF4G2 | eukaryotic translation initiation factor 4 gamma 2 [Source:HGNC Symbol;Acc:HGNC:3297] | 11313 | -0.473 | -0.1725 | No |
| 59 | HLA-E | "major histocompatibility complex, class I, E [Source:HGNC Symbol;Acc:HGNC:4962]" | 11659 | -0.526 | -0.1729 | No |
| 60 | EIF4G1 | eukaryotic translation initiation factor 4 gamma 1 [Source:HGNC Symbol;Acc:HGNC:3296] | 12122 | -0.625 | -0.1776 | No |
| 61 | ABL2 | "ABL proto-oncogene 2, non-receptor tyrosine kinase [Source:HGNC Symbol;Acc:HGNC:77]" | 12342 | -0.688 | -0.1602 | No |
| 62 | HLA-A | "major histocompatibility complex, class I, A [Source:HGNC Symbol;Acc:HGNC:4931]" | 12502 | -0.749 | -0.1350 | No |
| 63 | HLA-B | "major histocompatibility complex, class I, B [Source:HGNC Symbol;Acc:HGNC:4932]" | 12534 | -0.764 | -0.0991 | No |
| 64 | ACTG1 | actin gamma 1 [Source:HGNC Symbol;Acc:HGNC:144] | 12613 | -0.806 | -0.0648 | No |
| 65 | HLA-DRB4 | "major histocompatibility complex, class II, DR beta 4 [Source:HGNC Symbol;Acc:HGNC:4952]" | 12636 | -0.815 | -0.0257 | No |
| 66 | ACTB | actin beta [Source:HGNC Symbol;Acc:HGNC:132] | 12757 | -0.920 | 0.0111 | No |
Table: GSEA details [plain text format]

  

Fig 2: KEGG\_VIRAL\_MYOCARDITIS      
 Blue-Pink O' Gram in the Space of the Analyzed GeneSet

  

Fig 3: KEGG\_VIRAL\_MYOCARDITIS: Random ES distribution      
 Gene set null distribution of ES for **KEGG\_VIRAL\_MYOCARDITIS**
